# Correlation of Artemin and GFRα3 With Osteoarthritis Pain: Early Evidence From Naturally Occurring Osteoarthritis-Associated Chronic Pain in Dogs

**DOI:** 10.3389/fnins.2020.00077

**Published:** 2020-02-13

**Authors:** Laura Minnema, Joshua Wheeler, Masataka Enomoto, Saumitra Pitake, Santosh K. Mishra, B. Duncan X. Lascelles

**Affiliations:** ^1^Translational Research in Pain Program, Department of Clinical Sciences, College of Veterinary Medicine, North Carolina State University, Raleigh, NC, United States; ^2^Department of Molecular Biomedical Sciences, College of Veterinary Medicine, North Carolina State University, Raleigh, NC, United States; ^3^Comparative Pain Research and Education Center, College of Veterinary Medicine, North Carolina State University, Raleigh, NC, United States; ^4^Thurston Arthritis Research Center, UNC School of Medicine, Chapel Hill, NC, United States; ^5^Center for Translational Pain Research, Department of Anesthesiology, Duke University, Durham, NC, United States

**Keywords:** osteoarthritis, pain, DRG, GDNF, artemin, GFRα3, dogs, human

## Abstract

Arthritis, including osteoarthritis (OA) and other musculoskeletal-associated pain, is a worldwide problem, however, effective drug options are limited. Several receptors, neurotransmitters, and endogenous mediators have been identified in rodent models, but the relevance of these molecules in disease-associated pain is not always clear. Artemin, a neurotrophic factor, and its receptor, glial-derived neurotrophic factor (GDNF) family receptor alpha-3 (GFRα3), have been identified as involved in pain in rodents. Their role in OA-associated pain is unknown. To explore a possible association, we analyzed tissue from naturally occurring OA in dogs to characterize the correlation with chronic pain. We used behavioral assessment, objective measures of limb use, and molecular tools to identify whether artemin and GFRα3 might be associated with OA pain. Our results using banked tissue from well-phenotyped dogs indicates that artemin/GFRα3 may play an important, and hitherto unrecognized, role in chronic OA-associated pain. Elevated serum levels of artemin from osteoarthritic humans compared to healthy individuals suggest translational relevance. Our data provide compelling evidence that the artemin/GFRα3 signaling pathway may be important in OA pain in both non-humans and humans and may ultimately lead to novel therapeutics.

## Introduction

A significant proportion of the economic burden of chronic pain is due to musculoskeletal pain, and yet treatment options remain limited and insufficient for the alleviation of osteoarthritis (OA)-associated pain, the major component of musculoskeletal pain (Burgess and Williams, 2010). Despite significant successes arising from basic pain research in terms of our understanding of molecular mechanisms and the growing number of potential targets for new drug development, drug candidates are failing when they enter Phase II (efficacy) trials in humans due to limited knowledge of the cellular and molecular components involved in disease conditions. Diseases in companion animals have been suggested to be good models of certain human conditions, including OA (Mogil, 2009; Percie du Sert and Rice, 2014; Klinck et al., 2017; Lascelles et al., 2018).

The glial cell line-derived neurotrophic factor (GDNF) family members (GDNF, neurturin, and artemin) sensitize nociceptors; in particular, artemin is an important effector in inflammatory hyperalgesia (Malin et al., 2006). Artemin is thought to be an important endogenous mediator for inflammation, migraine, burning mouth syndrome, and neuropathic cold allodynia (Lippoldt et al., 2013, 2016; Shinoda et al., 2015; Shang et al., 2016; Nencini et al., 2018). Recently, artemin signaling pathways have been shown to be involved in the pathogenesis of bone pain (Nencini et al., 2018). Another important role of artemin has been identified in the trigeminal ganglia (TG), where artemin regulates an inducible form of nitric oxide synthase (iNOS). The regulation of iNOS might be involved in the mechanism through which artemin participates in the trigeminal pain pathways (Shang et al., 2017). Furthermore, anti-artemin therapy has been shown to be effective in preventing and reversing ongoing bladder hyperalgesia in an animal model of cystitis (DeBerry et al., 2015). In contrast, artemin exerts an antinociceptive effect on herpes-related pain by modulating the dynorphin levels in the central nervous system of HSV-inoculated mice (Asano et al., 2006). Based on this evidence, peripheral artemin is involved in generating pain signals in rodents.

The GDNF family of ligands mediate their effects through GDNF receptors, and, at this point, four different members of this family have been identified (GFRα1-4). GDNF binds to GFRα1, neurturin activates the GFRα2 receptor, and artemin binds to the GFRα3 receptor. All of the GDNF-receptors form a complex with the tyrosine kinase RET, and then, through RET, activate several intracellular signaling pathways (Baloh et al., 1998a, b). GFRα1 and GFRα2 receptor expression has been demonstrated in the non-peptidergic neuronal populations, but, in contrast, GFRα3 is predominantly expressed by a subpopulation of nociceptive sensory neurons that are peptidergic (Orozco et al., 2001; Elitt et al., 2006), some or all of which also express the Ret receptor tyrosine kinase, and by the transient receptor potential (TRP) ion channel proteins TRPV1 and TRPA1 (Orozco et al., 2001; Elitt et al., 2006; Goswami et al., 2014). Furthermore, the GFRα3 receptor is also expressed by transient receptor potential cation channel subfamily melastatin (TRPM8) expressing neurons that are involved in encoding cold sensations (Lippoldt et al., 2013, 2016). Outside of the dorsal root ganglia (DRG), other reports suggest the expression of GFRα3 in non-neuronal cells (Yang et al., 2006; Thai et al., 2019). Another receptor sub-family, GFRα4, is not functionally expressed in the DRG (Lindahl et al., 2000; Luo et al., 2007). All evidence considered, it is clear that GFRα3 is exclusively expressed in nociceptive neurons in the DRG, suggesting that artemin/GFRα3 interaction is important in the transmission of pain and could be a potential target in the development of analgesia.

Overall, artemin/GFRα3 is involved in inflammation and bone pain in rodents, but its expression and functional correlation with pain in naturally occurring OA are unknown, to the best of our knowledge. By analyzing samples from dogs with naturally occurring OA, we aimed to identify relevant and translationally resilient pharmacological target(s). We employed this innovative approach to identify whether artemin/GFRα3 may play a role in OA-associated pain. We analyzed DRG from pet dogs with naturally occurring OA and found significantly increased GFRα3 in the DRG serving osteoarthritic joints. This led us to hypothesize that the expression of its ligand, artemin, would be increased in serum and that synovial fluid artemin may be related to joint pain in dogs with OA. Next, we explored the relationship between serum artemin and OA status in humans. In summary, our results provide the first evidence of a link between artemin/GFRα3 and OA-associated pain in the natural disease state.

## Materials and Methods

### Collection of Tissues and Fluids

The serum and synovial fluid samples used in the work described here were collected from client-owned pet dogs that had been carefully evaluated and found to have OA-associated pain or found to be normal (i.e., pain-phenotyped animals), as a result of their involvement in veterinary clinical research studies (Muller et al., 2019) or ongoing translational pain research studies at NC State College of Veterinary Medicine. The breeds of dogs included were representative of the medium to large breed dogs that present for OA-associated pain. None of these dogs were receiving or had received analgesics for 4 weeks prior to sample collection. All dogs had undergone radiographic evaluation and examination by a specialist veterinary orthopedic surgeon and had clinical metrology instrument data available to define whether OA was present and whether pain was associated with it. In addition, these data were supported in some cases by force plate data on limb use. The characteristics of dogs used in various parts of this work are detailed in Table 1.

**TABLE 1:** Demographic and pain status characteristics of dogs used in the various experiments reported.

	OA dogs^a^, serum artemin (*n* = 25)	Healthy dogs^a^, serum artemin (*n* = 11)	OA dogs^b^, synovial fluid artemin (*n* = 8)	OA dogs^c^, Dorsal root ganglia
Age, years (mean ± SD, range)	8.6 ± 1.97; 4 to 12	3.73 ± 2.02; 1 to 8	8.03 ± 4.26; 1.2 to 13	3.43 ± 0.53; 3 to 4
Sex	13 MC; 12 FS	7 MC; 4 FS	4 MC; 3FS; 1M	7 MC
Weight, kg (mean ± SD, range)	27.92 ± 7.74; 16.7 to 44.8	26.37 ± 7.50; 15.8 to 40.5	37.12 ± 5.95; 27.1 to 45.2	28.2 ± 1.9; 25 to 30
Body Conditions Score [(BCS) median, range]	5 (5 to 8)	5 (5 to 8)	5; 5 to 8	5; 5 to 6
LOAD score (mean ± SD, range)	20.24 ± 7.22; 10 to 38	3.63 ± 3.23; 0 to 9	23.3 ± 6.88; 14 to 36	
CBPI pain score (mean ± SD, range)	3.45 ± 1.55; 1 to 5.75	0, 0	4.83 ± 1.84; 2 to 7.75	
CBPI interference score (mean ± SD, range)	3.63 ± 2.00; 1 to 8.33	0, 0	5.2 ± 1.78; 2.5 to 7.33	
Joints affected with OA-associated pain* (left and right included as separate entries); OR, Index joints (synovial fluid)	Carpus 12; Elbow 27; Shoulder 5; Tarsus 1; Stifle 6; Hip 40	None	Stifle (3); elbow (4); hock (1)	Unilateral hip
SI PVF (mean ± SD, range)			−22.3 ± 10.4; −40.3 to −11.8	−6.89 ± 4.47; −13.54 to −2.74
SI VI (mean ± SD, range)			−17.7 ± 10.9; −35.7 to −5.2	−5.31 ± 10.10; −21.50 to 3.40

*OA dogs had multiple joints affected by OA and pain. Serum and synovial fluid samples were procured from different cohorts of dogs: ^*a*^Samples taken from pet dogs at screening prior to clinical study (Muller et al., 2019); ^*b*^Samples taken from pet dogs in pilot study prior to intra-articular injection of an experimental therapeutic (unpublished); ^*c*^Samples taken at euthanasia of research dogs with unilateral OA.

Peripheral nervous system tissue samples (lumbar DRG) were collected after euthanasia from well-phenotyped research animals of known pain and OA status [similar to a previous study (Little et al., 2016)]. These were mixed-breed hounds, and they had not received any analgesic treatment. DRG (L4-L6) samples were collected within 30 min of euthanasia, with DRG identified by counting lumbar vertebrae. All these dogs had radiographic confirmation of OA and force plate data verifying unilateral joint pain (Table 1, column 4). DRG samples for the pilot data (not shown here) were collected from pet client-owned dogs being euthanized due to unilateral OA pain that were examined and evaluated by a veterinarian prior to euthanasia.

All original studies and sample collection were conducted with informed and written owner consent and Institutional Animal Care and Use Committee (IACUC) approval. All samples were stored at −80°C within 2 h of collection, or 2 h of euthanasia in the case of DRG, and all the samples to be used in this study were collected within the last 5 years.

### Measurement Ground Reaction Forces

Kinetic gait analysis of limb use was performed using dual in-series force plates (ATMI, Watertown, MA, United States). Dogs (Table 1, column 3) were specifically recruited for having single limb lameness, as demonstrated by a lower peak vertical force [(PVF) expressed as a percentage of bodyweight] compared to the contralateral limb, detectable pain on manipulation, and radiographic evidence of OA in one joint in the index limb. Dogs were trotted over the force plates, and trials were accepted as valid if the dog trotted across the force plate in a straight line and an observer confirmed foot strikes on the plates without noting any unusual activity by the dog (pulling, visually detectable movement of the head from side to side), with a target velocity of 1.7–2.1 m/s and within acceleration changes of ±0.5 m/s^2^. Gait velocity and acceleration were measured by means of five photometric switches (photocells 50 cm apart) connected to the computer analysis system. Five valid trials were collected from each dog during trotting, and data were collected for all limbs. A single handler and observer performed all gait analysis. Specialized computer software (Sharon software, DeWitt, MI, United States) was used to calculate the ground reaction forces (GRFs) of the limbs. GRFs were expressed in percent body weight, and then symmetry indices were calculated for the index limb and the contralateral limb. Symmetry indices (SI) for PVF and vertical impulse (VI) were calculated by the use of the following equation.

$$S\,I=\frac{\left(x_{i}-x_{c}\right)}{\left(1/2\right)\,\left(x_{i}+x_{c}\right)}\times 100$$

where *x*_*i*_ is the mean of a given gait variable for the index limb, and *x*_*c*_ is the mean of a given gait variable for the contralateral limb. By using symmetry indices, data for fore and hind limbs can be combined.

### Clinical Metrology Instrument Assessments of Pain and Mobility Impairment

Where applicable, caregiver (owner) assessments of pain, activity, and function were captured using validated clinical metrology instruments (questionnaires): the Liverpool Osteoarthritis in Dogs (LOAD) (Hercock et al., 2009; Walton et al., 2013) and the Canine Brief Pain Inventory (CBPI) (Brown et al., 2007). The LOAD is a 13-item instrument with all items reported on a five-point Likert-type scale. Each item is scored between 0 and 4, and the item scores are summed to give an overall instrument score. The LOAD covers three domains: activity/exercise, stiffness/lameness, and the effect of weather (Walton et al., 2013). The CBPI is a two-part instrument based on the human Brief Pain Inventory; the pain severity score (CBPI PSS) is the arithmetic mean of four items scored on an 11-point (0 to 10) numerical scale, and the pain interference score (CBPI PIS) is the mean of six items scored similarly. It has been reported to measure two dimensions (pain and interference) (Walton et al., 2013).

### Quantitative RT-PCR

Total RNA was extracted from DRG using Qiagen’s RNAeasy kit and converted into cDNA. Quantitative real-time PCR was accomplished using commercially available TaqMan primer sets. Samples were run in triplicate. Amplification efficiencies were normalized against GAPDH, a standard housekeeping gene (Wheeler et al., 2019). Individual C_*T*_ values were calculated using StepOne Software v2.2.2. Relative expression (ΔC_*T*_) of each gene was calculated with the following equation: Δ*C*_*T*_=*C*_*T*,*GAPDH*_-*C*_*T*,*GOI*_, where *GOI* is the gene of interest. ΔC_*T*_ values were linearized using 2^−Δ*C*_*T*_^ and then multiplied by 1000 so that the y-axis is on a more intuitive scale. Standard deviation was calculated using the linearized ΔC_*T*_ values and used in the SEM calculation: S⁢E⁢M=standard⁢deviation/tn⁢e⁢c⁢h⁢n⁢i⁢c⁢a⁢l, where *n*_*technical*_ is the number of technical replicates. The Taqman probes for the dog genes studied were purchased from Thermofisher, Carlsbad, CA, United States: TRPV1 (Cf027233943), GFRα3 (Cf02675182), and GAPDH (Cf04419463).

### Western Blot

To extract total protein, DRG were homogenized using a tissue homogenizer in the presence of 100 μl of ice-cold RIPA buffer supplemented with protease inhibitor tablets ı(Pierce^TM^). Total protein of lysates was measured using standard BCA (Bicinchoninic Acid Assay). Protein lysates were then denatured by heating at 95°C in Laemmli’s buffer containing 2% w/v SDS, 62.5 mM Tris (pH 6.8), 10% glycerol, 50 mM DTT, and 0.01% w/v bromophenol blue (Pitake et al., 2019). The lysates were cooled on ice and briefly micro-centrifuged. Aliquots of 35 μg of DRG protein were loaded onto a 4–12% SDS-PAGE gel, and subsequently electro-blotted onto PVDF membranes. Membranes were incubated in 15 ml of blocking buffer (20 mM Tris base and 140 mM NaCl, 5% bovine serum albumin, and 0.1% Tween-20) for 1 h. Membranes were then incubated with the desired primary antibody [rabbit anti-GFRα3 primary antibody (RA30017), 0.2 μg/ml; loading control mouse anti-GAPDH (Cat. sc-32233)] diluted in 10 ml of blocking buffer at 4°C overnight. The next day, the membrane was washed and incubated with an appropriate horseradish peroxidase-conjugated secondary antibody [secondary goat anti-rabbit-HRP from Santa Cruz (sc-2030); secondary goat anti-mouse IgG HRP) (1:1000)] in 10 ml blocking buffer for 1 h at room temperature. Immuno-reactive proteins were revealed using enhanced chemiluminescence detection (Pierce ECLTM). Densitometry analysis was performed using open-source ImageJ software from NIH.

### Quantitative Measurement of Artemin

Artemin was analyzed using a quantitative competitive enzyme-linked immunosorbent assay (ELISA). Canine serum samples from OA dogs and normal dogs (*n* = 25 OA; *n* = 11 normal) were stored at −80°C and thawed and vortexed before use. Canine serum samples were run according to the protocol provided by the kit (ABclonal Canine Artemin ELISA Kit CA0039). The stop solution changes color from blue to yellow; the intensity of the color was measured at 450 nm using a spectrophotometer (Labsystems Fluoroskan Ascent FL ELISA plate reader) and sample concentrations were determined by comparison to the standard curve.

Artemin levels from human serum were measured using the R&D Systems Human Artemin DueSet ELISA (R&D, DY2589) and the associated protocol. Human serum samples were obtained from Reprocell (Beltsville, MD) after searching their database of available samples. All OA serum samples (*n* = 5) were from middle-aged (45–63), female patients who had endured chronic, multiple-joint OA pain for a minimum of 13 years. Non-OA control serum samples (*n* = 4) were obtained from the same company and matched for age and gender. Serum samples were stored at −80°C until used, at which point they were vortexed and aliquoted for the ELISA. All samples were run in triplicate. Spectrophotometer readings were at 450 and 570 nm. The 570 nm readings were subtracted from the 450 nm for the most accurate results. Sample concentrations were determined by comparison to the standard curve.

### Statistical Approach Including Sample Size Estimations

Data are expressed as mean ± SEM. All assays were performed by individuals who were blinded to the metadata. Statistical analysis was performed in GraphPad Prism and JMP statistical software. For canine serum artemin, differences between the two groups were examined using a parametric Student’s *t*-test, with *p* < 0.05 considered significant and *p* > 0.05 considered non-significant. A formal sample size estimation was used for serum artemin concentrations, based on pilot data, and indicated that a total sample size of 28 would be required for a power of 0.8, at an alpha of 0.05. Canine serum artemin concentrations were compared to force plate data (symmetry indexes) and owner questionnaires using linear regression. Correlation coefficients (R-squared values) and *p*-values were generated using JMP-Pro software. The threshold to determine whether a correlation was significant was set at a *p*-value of 0.05. Similar sample size calculations for the RT-PCR pilot data indicated that a paired sample size of 7 was needed for a power of 0.8 at an alpha of 0.05. No other sample size estimations were performed. For Western blot assay, we performed a pairwise *t*-test and determined the statistical significance. Sample size for human serum samples was limited due to the availability of the samples; a 2-tailed Student’s *t*-test was used to evaluate the data statistically. Data were tested for normal or non-normal distribution, and appropriate statistical tests were used. Animals and data points were not excluded from the analysis. All relevant data are available from the authors.

## Results

### DRG Sensory Neurons Express GFRα3

**FIGURE 1:**
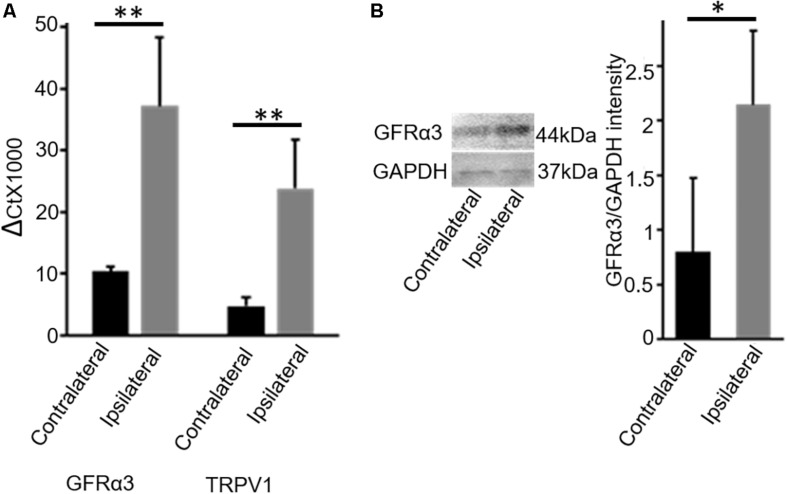
DRG tissue of osteoarthritis dogs shows an increase in expression of GFRα3-receptors. **(A)** Quantitative PCR was used to quantify the expression of *Gfr*α*3* and *Trpv1* genes relative to glyceraldehyde-3-phosphate dehydrogenase (GAPDH) in contralateral (black) and osteoarthritis ipsilateral (gray) DRG. An approximately 3.5-fold change in GFRα3 expression compared to the contralateral side was observed in osteoarthritis ipsilateral DRM, at a level comparable with *Trpv1*, a well-known pain receptor for thermal pain. Data represent mean ± SEM for cDNA preparations *n* = 7. Significance was determined by Student’s *t*-test, *p* ≤ 0.05; ^∗∗^*p* ≤ 0.01. **(B)** Western blotting was used to quantify the expression of GFRα3-receptor at the protein level, and we found that osteoarthritis dogs (ipsilateral; gray) have an approximately 3-fold increase in GFRα3 receptor protein relative to the contralateral side (black). Data represent mean ± SEM for protein detection, *n* = 6. Significance was determined by using pairwise *t*-test, ^∗^*p* ≤ 0.05.

In early pilot work, we evaluated dog DRG tissue for multiple receptors that we had found to be expressed on TRPV1-expressing neurons in the dorsal root ganglia (DRG) in mice (Goswami et al., 2014). This pilot work indicated increased GFRα3 in DRG serving osteoarthritic joints in dogs. Therefore, we determined the expression levels of GFRα3 in the lumbar (L4-L6) DRG serving contralateral versus ipsilateral joints with associated pain within the same dog (*n* = 7; Table 1, column 4) by using quantitative RT-PCR. Our results showed an approximately 4-fold increase in GFRα3 expression in ipsilateral DRG serving OA joints compared to those serving normal joints (Figure 1A) suggesting a role of GFRα3 in OA pain. We also compared the expression of TRPV1 receptors, a well-known molecule involved in pain signal transduction (Mishra et al., 2011). Our results demonstrated an increase in the expression of *Trpv1* mRNA in the ipsilateral DRG compared to contralateral from OA dogs.

Next, we examined the expression of GFRα3 at the protein level in the DRG sensory neurons. Due to an absence of a dog-specific GFRα3 antibody and because of high homology between the dog and mouse protein sequences (76% identity) for the GFRα3 receptor, we used an anti-mouse GFRα3 antibody for the Western blotting. In addition to overall sequence homology, the synthetic peptide corresponding to amino acids from 347 to 360 of the murine GFRα3 has 90% identity with the dog-predicted GFRα3 receptor. We performed GFRα3 antibody characterization using negative control (heart tissue) and secondary antibody control (data not shown). However, these methods still do not validate the specificity of the antibody, which needs to be further tested either in GFRα3 knockout mice or using pre-adsorption with GFRα3 synthetic protein/peptide. We used Western blotting to detect and quantify the presence of GFRα3 receptors in the DRG serving contralateral versus painful OA (ipsilateral) joints within the same dog. We identified GFRα3 protein in DRG from contralateral DRGs and found an increase in GFRα3-receptor protein in the ipsilateral OA DRGs, again suggesting a role of GFRα3 in pain sensitivity (Figure 1B).

### Upregulation of Serum Artemin in Osteoarthritic Dogs

**FIGURE 2:**
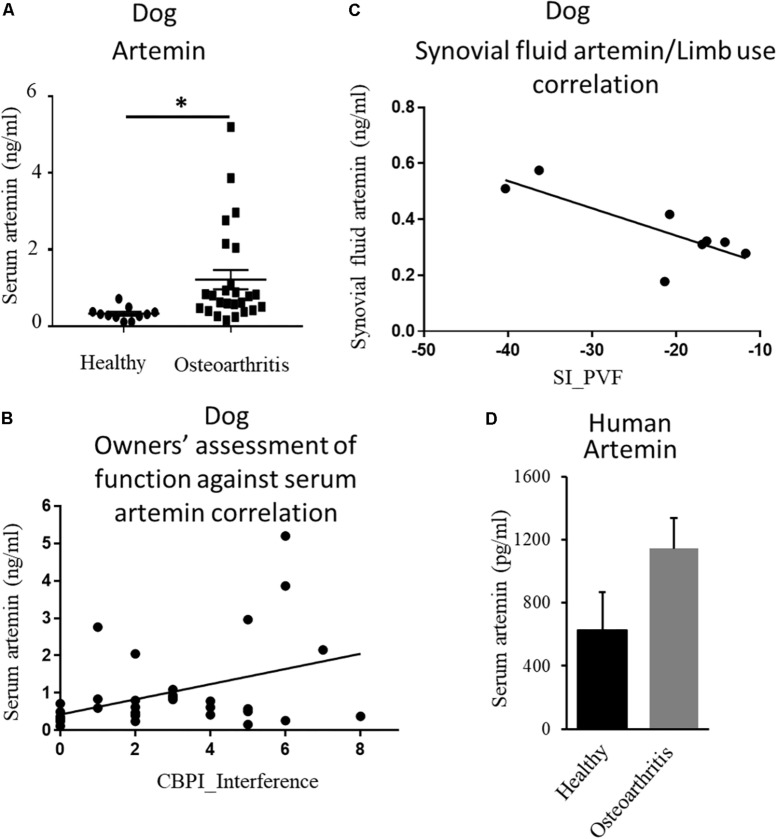
Endogenous ligand artemin and its correlation with pain. **(A)** Quantification of serum artemin concentrations (ng/mL) in healthy dogs (*n* = 11) and the dogs with OA-associated pain (*n* = 26) using ELISA. Data are represented as mean ± SEM. Significance was determined by using Student’s *t*-test, ^∗^*p* ≤ 0.05. **(B)** Plot of serum artemin concentrations (ng/mL) against owner-assessed disability (CBPI interference score), showing that increased serum artemin concentrations are associated with greater disability in dogs (*R*^2^ = 0.16; *p* = 0.014). **(C)** Plot of synovial fluid artemin concentrations (ng/mL) against limb use (*n* = 8), expressed as a Symmetry Index (SI) of peak vertical force. Negative values of SI correspond to decreased limb use, and the plot shows that increased synovial fluid concentrations of artemin correspond to less limb use (*R*^2^ = 0.62; *p* = 0.02). **(D)** Human serum samples obtained from OA and healthy individuals were measured using ELISA. Data are represented as mean ± SEM (non-OA control, *n* = 4; OA, *n* = 5; *p* = 0.1). *P*-value was determined via 2-tailed *t*-test.

The endogenous ligand for GFRα3 is artemin (Baloh et al., 1998a, b; Wang et al., 2006). Having found increased expression of GFRα3, we investigated whether circulating or local levels of artemin were increased in dogs with painful OA. Serum samples were collected from non-OA healthy controls and dogs with OA-associated pain at the time of screening, prior to their involvement in a clinical study (Muller et al., 2019). The demographics of the dogs are tabulated in Table 1 (columns 1 and 2). The OA dogs were older, but otherwise there were no differences between the groups. We measured serum artemin concentrations using ELISA, which showed an increased concentration of artemin in dogs with OA-associated pain compared to pain-free healthy dogs (Figure 2A). Pain and disability in pet dogs with OA can be measured using validated clinical metrology instruments (CMIs), and we found that higher serum artemin concentrations were associated with higher scores on the CMIs and thus greater levels of pain and functional impairment. There was a significant relationship between LOAD index values and serum artemin concentrations (*R*^2^ = 0.11; *p* = 0.05) and significant relationships between serum artemin and the Canine Brief Pain Inventory (CBPI) pain (*R*^2^ = 0.11; *p* = 0.049) and CBPI interference (*R*^2^ = 0.16; *p* = 0.014) sub-scales (Figure 2B).

Next, we evaluated local concentrations of artemin in synovial fluid and compared synovial fluid artemin concentrations with objectively measured limb use (a measure of joint pain). Synovial fluid samples were collected from dogs enrolled in a pilot study aimed at assessing the efficacy of a TNFα fusion protein injected intra-articularly (unpublished data). The demographics of the dogs are tabulated in Table 1. Synovial fluid artemin concentrations were normally distributed. There was a significant, negative relationship between synovial fluid levels and index limb use compared to contralateral limb, expressed as the symmetry index of PVF (*R*^2^ = 0.62; *p* = 0.02) (Figure 2C), and a similar pattern was seen for the symmetry index of vertical impulse (VI) (Supplementary Figure S1).

### Upregulation of Serum Artemin in Osteoarthritic Human Serum

After finding upregulation of artemin in canine serum samples, we investigated whether this relationship held true in humans. Human serum samples were obtained from patients with well-characterized OA and from age- and sex-matched controls (Reprocell, Beltsville, MD). Samples were from female patients who had been suffering from OA for a minimum of 13 years (*n* = 5) and age/gender-matched controls (*n* = 4). Samples were analyzed for artemin with an ELISA. Our results showed a trend of an increased level of artemin in people with OA-associated pain compared to pain-free healthy individuals (Figure 2D, *p* = 0.1).

## Discussion

OA patients experience chronic pain, however, the molecular basis for chronic pain in these patients is still largely unknown (Eitner et al., 2017). Although it has recently been revealed that neurotrophic factors play an important role in pain transduction by activating the receptors present on the DRG sensory neurons in mice (Orozco et al., 2001; Elitt et al., 2006; Malin et al., 2006; Kashyap et al., 2018; Nencini et al., 2018), it is currently unknown whether artemin, an endogenous neurotrophic factor, and its receptor, GFRα3, play an important role in pain hypersensitivity in OA patients. Here, we combined molecular and immunological methods to explore the association of artemin and GFRα3 with OA-associated pain in dogs with OA. Using within-dog control tissues, we found increased expression of GFRα3 in DRG serving painful OA joints. We found a positive association between peripherally released artemin in the synovial fluid and joint pain, as measured by limb use. Although the work with both DRG and synovial fluid only involved small numbers of dogs, it does suggest that the artemin/GFRα3 axis is upregulated in OA pain states. Also, we found an association between serum artemin concentrations and OA, both in dogs and in humans. The positive correlation between artemin concentrations and owner questionnaire scores (LOAD and CBPI), albeit a weak correlation, is interesting. The correlations between the questionnaires and serum artemin were low but are most significant for the CBPI pain and interference subscales. The CBPI focuses primarily on pain and the impact of pain on the ability to perform activities. The relationship between serum artemin and such scores should not be interpreted as evidence of a serum biomarker but rather as additional evidence of a potential role for artemin in the OA pain state. It must be cautioned that our data only show a correlation or association between OA pain and artemin/GFRα3, not causation. Regardless, we believe these data form a compelling rationale for investigating the role of artemin/GFRα3 in OA pain in rodent OA models.

Initially, glial cell line-derived neurotrophic factors (GDNFs), such as artemin, were studied for their role in development and neuronal survival (Luo et al., 2007), but recently there has been a shift to examining their role in the modulation of pain. There is already evidence that artemin and GFRα3 have a role in pain in rodents. Several researchers have found increased levels of artemin associated with tissue damage or inflammation in rodent pain models, such as CFA injection (Malin et al., 2006; Ikeda-Miyagawa et al., 2015; Lippoldt et al., 2016) and the nitroglycerin (NTG) migraine model (Shang et al., 2016). GFRα3 expression has also been tied to painful conditions such as cold allodynia and thermal hypersensitivity (Malin et al., 2006; Lippoldt et al., 2016). Other data indicate that GFRα3 knockout mice do not acquire the hypersensitivity normally seen with CFA injection, nerve injury, or chemotherapy (Lippoldt et al., 2016). GDNF, a ligand in the same family as artemin, has been implicated in pain sensitivity in dogs in a study by Plassais et al. (2016), which found that a mutation that decreased the expression of GDNF was responsible for pain insensitivity in dogs with self-mutilation syndrome. There has recently been an investigation into the role of GDNFs in inflammatory bone pain using an acute CFA-induced model in rats. The investigators found that artemin was able to sensitize bone afferent neurons to mechanical stimulation, but they found no upregulation of GFRα3 (Nencini et al., 2018).

Part of the interest in the role of GFRα3 in pain comes from its co-localization and interactions with the TRPV1 ion channel. TRPV1 and GFRα3 are highly co-localized, and knockdown of GFRα3 results in a lack of axotomy-induced increase in TRPV1 expression (Jankowski et al., 2010). Further, investigations have shown that artemin injections can induce the upregulation of TRPV1 (Ikeda-Miyagawa et al., 2015) and can sensitize TRPV1, producing heat hyperalgesia (Malin et al., 2006). Interestingly, our results suggest similar findings, with GFRα3 expression increases occurring in conjunction with TRPV1 expression in dog DRG as measured by qPCR. In the future, it will be interesting to determine the different subpopulations of neuronal types in the dog DRG by developing some canine-specific antibodies, which will provide information about various subsets of neurons that have been identified in mouse DRG.

The exact cellular mechanisms for artemin’s potential actions in OA have not been investigated. OA is a disease characterized by hypersensitivity to hot, cold, and mechanical pain (Arendt-Nielsen et al., 2018), and this hypersensitivity is driven by neuronal changes in the DRG. This may be mediated in part by GFRα3. Data suggest that artemin/GFRα3 is upstream in a pathway that regulates TRP channels (Ikeda-Miyagawa et al., 2015). The GFRα3 signaling pathway acts through the activation of the tyrosine kinase RET, possibly the RET51 isoform, and downstream effects may be due to the ERK/MAPK pathway (Li et al., 2009). A role in the development and maintenance of OA pain and sensitivity, possibly through the ERK/MAPK pathway, would fit in with our current knowledge of GFRα3/artemin. However, this needs to be investigated in the future.

Next, our results indicate that artemin/GFRα3 may play a hitherto unrecognized role in OA-associated pain and hypersensitivity. We have shown that artemin is related to various measures of OA-associated pain, which suggests a possible mechanism for broad thermal and mechanical hypersensitivity in OA patients. By using samples from naturally occurring OA in dogs, we were able to identify this potential target in the natural disease state. There is growing interest in this general approach—so-called reverse translation or multidirectional translation—with neurobiological evidence from the target condition being used to validate the model and inform mechanistic research (Dawes et al., 2011, 2014). Companion animals are particularly useful in this regard, as they share the same environment and habits as their human owners, making them ideal for investigations into diseases that affect both species, such as osteoarthritis. Future research will determine whether such a reverse translational approach is a good way to identify relevant targets for mechanistic evaluation in rodent models.

Osteoarthritis in pet dogs is very similar to human OA [biomechanically, structurally, histologically, genomically, and molecularly (Clements et al., 2006; Proffen et al., 2012; McCoy, 2015)], and recent reviews have highlighted the potential of using pet dogs to inform the translational process, particularly for efficacy screening of putative analgesics prior to human clinical trials (Klinck et al., 2017; Lascelles et al., 2018). It has been proposed that using tissue from naturally occurring disease states will better inform the direction of basic research and the development of novel targets (Klinck et al., 2017; Lascelles et al., 2018). Interestingly, during the drafting of this manuscript, Regeneron announced that it was advancing a fully human antibody to the GFRα3 neurotrophic factor receptor into clinical studies for OA pain in humans. Some limitations of this study include a small sample size of human serum samples and an age discrepancy between the control and OA dogs. Our study highlights a clinically relevant avenue for further research to determine the role of artemin/GFRα3 in OA pain.

## Data Availability Statement

All datasets generated for this study are included in the article/Supplementary Material.

## Ethics Statement

The animal study was reviewed and approved by the North Carolina State University Institutional Animal Care and Use Committee (IACUC). All original studies and sample collection were conducted under-informed and written owner consent and IACUC approval. Written informed consent was obtained from the owners for the participation of their animals in this study. Protocols used under this study includes: Dog Studies – 13-010-B, 15-163-O, and 16-094.

## Author Contributions

BL and SM conceived the idea and designed the experiments. LM, JW, ME, and SP performed the experiments. LM, JW, BL, and SM analyzed the data. LM, BL, and SM wrote the manuscript.

## Conflict of Interest

The authors declare that the research was conducted in the absence of any commercial or financial relationships that could be construed as a potential conflict of interest.

## Supplementary Material

FIGURE S1 Correlations of artemin and limb use during pain. Plot of synovial fluid artemin concentrations (ng/ml) against limb use, expressed as a symmetry index (SI) of vertical impulse (VI). Negative values of SI correspond to decreased limb use, and the plot shows that increased synovial fluid concentrations of artemin correspond to less limb use (*R*^2^ = 0.44; *n* = 8; *p* = 0.074).
