# Supplementary figures and images for: Correlation of Artemin and GFRα3 With Osteoarthritis Pain: Early Evidence From Naturally Occurring Osteoarthritis-Associated Chronic Pain in Dogs
Source: Front Neurosci. 2020 Feb 13;14:77. doi: 10.3389/fnins.2020.00077 (PMC7031206; doi:10.3389/fnins.2020.00077)

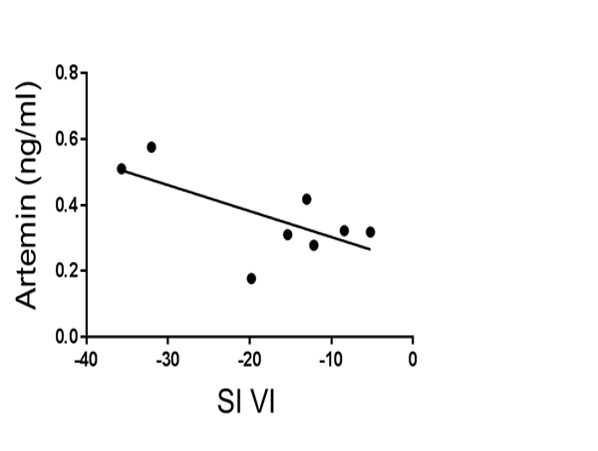

Supplement: FIGURE S1 — Correlations of artemin and limb use during pain. Plot of synovial fluid artemin concentrations (ng/ml) against limb use, expressed as a symmetry index (SI) of vertical impulse (VI). Negative values of SI correspond to decreased limb use, and the plot shows that increased synovial fluid concentrations of artemin correspond to less limb use (R2 = 0.44; n = 8; p = 0.074). [file Image_1.tif]
